# The use of *Andrographis paniculata* and its effects on liver biochemistry of patients with gastrointestinal problems in Thailand during the COVID-19 pandemic: a cross sectional study

**DOI:** 10.1038/s41598-022-23189-7

**Published:** 2022-10-29

**Authors:** Apichat Kaewdech, Siwanon Nawalerspanya, Suraphon Assawasuwannakit, Naichaya Chamroonkul, Sawangpong Jandee, Pimsiri Sripongpun

**Affiliations:** 1 Gastroenterology and Hepatology Unit, Division of Internal Medicine, Faculty of Medicine, Prince of Songkla University, Songkhla, Thailand; 2 Department of Internal Medicine, Phaholponpayuhasena Hospital, Kanchanaburi, Thailand; 3 Department of Medicine, Panyananthaphikkhu Chonprathan Medical Center, Srinakharinwirot University, Nonthaburi, Thailand

**Keywords:** Hepatitis, Patient education

## Abstract

In the COVID-19 pandemic, healthcare facility supply and access are limited. There was an announcement promoting *Andrographis paniculata* (ADG) use for treatment of mild COVID-19 patients in Thailand, but misconception of taking for prevention might occur. Moreover, the effect of ADG on liver function test (LFT) has not been established. To study the ADG use and effect on LFT in patients with gastrointestinal (GI) problems, conducted a cross-sectional study including GI patients who voluntarily filled the ADG questionnaire in Aug–Sep 2021. LFT data at that visit and at the prior visit (if available) were obtained. The changes in LFT within the same person were analyzed and compared between patients with and without ADG consumption. During the study period, a total of 810 patients completed the survey, 168 patients (20.7%) took ADG within the past month. LFT data were available in 485 (59.9%) patients, the median alanine aminotransferase (ALT)  change compared with the prior visit was higher in the ADG vs control group (+ 2 vs 0, p = 0.029), and 44.5% had increased ALT (> 3 U/L) vs 32.2% in the ADG and control group, respectively (p = 0.018). Factors independently associated with an increased ALT, from a multivariable logistic regression, were ADG exposure (adjusted OR 1.62, p = 0.042), and patients with NAFLD who gained weight (adjusted OR 2.37, p = 0.046). In conclusion, one-fifth of GI patients recently took ADG, even it is not recommended for COVID-19 prevention. Those who took ADG are more likely to experience an increased ALT than who did not. The potential risk of ADG consumption on liver function should be further assessed.

## Introduction

The coronavirus disease 2019 (COVID-19) caused by the severe acute respiratory syndrome coronavirus 2 (SARS-CoV-2) is the current global pandemic, affecting the economy, social lifestyle, and people's health, and resulting in significant morbidity and mortality. As the pandemic continues and some new variants are associated with a rapid widespread of the disease, the healthcare system is greatly affected. In many countries, not only healthcare facilities but also medications to treat COVID-19 were in short supply. As a result, considerable attempts are being made to find novel treatment modalities, including herbal medicine, in addition to vaccination^1,2^.

*Andrographis paniculata* (ADG) or known as “King of bitters”, which has a major phytochemical constituent as Andrographolide, is a widely used ancient herbaceous plant that proposed to have various pharmacological effects including anti-pyretic, anti-inflammatory, analgesic or even hepatoprotectant^3–5^. ADG has many local names according to different areas of the world, e.g., “Kalmegh” in India, “Chuan-Xin-Lian” in China, “Senshinren” in Japan, “Green chiretta” in Scandinavian countries, or “Fah Talai Jone” in Thailand^6^. Recently, there have been published studies, both in vitro and in vivo, confirmed an anti-SARS-CoV-2 activity of ADG via a main mechanism of viral main protease binding site inhibitor. In brief, ADG can potentially inhibit viral replication, improve patient’s symptoms, reduce disease duration and severity in patients who exhibited mild symptom of COVID-19, nonetheless, ADG had no effect on viral entry process or prevention of SARS-CoV-2 infection^7–11^. The frequently reported side effects of ADG included nausea and vomiting, epigastric pain, diarrhea, chest pain, rash, and angioedema. Anaphylaxis or anaphylactic shock had been reported, there also were some reports regarding abnormal liver tests notably aspartate aminotransferase (AST) and/or alanine aminotransferase (ALT) elevation in some patients, but the data is scarce^10,12–16^. Surprisingly, although ADG has been used for a long time in many countries, there is limited data evaluating whether ADG has an impact on liver function tests (LFTs).

In Thailand, the COVID-19 situation was quite severe, like in many countries and causing antiviral medications shortage. With the anti-SARS-CoV-2 activity of ADG mentioned earlier, there was an announcement promoting ADG use to treat patients with confirmed SARS-CoV-2 infection who have mild symptoms of disease in Thailand. However, misconception of using ADG, not only for the treatment but also prevention of COVID-19, might occur on a public level, and it could lead to ADG misuse among healthy (non-SARS-CoV-2 infected) individuals. Moreover, it has been known that patients with pre-existing liver diseases, especially non-alcoholic fatty liver disease (NAFLD), might be at a higher risk to develop drug induced liver injury (DILI)^17–19^. Therefore, we conducted this study to evaluate the prevalence of ADG use among patients with gastrointestinal (GI) problems amid the pandemic of COVID-19 in Thailand and to determine the change in liver enzymes between those who had and did not have ADG consumption.

## Materials and methods

We conducted a single center cross-sectional study at our institute, which is a tertiary care university hospital in Southern Thailand. The study protocol was approved by the Institutional Human Research Ethics Committee (HREC), Faculty of Medicine, Prince of Songkla University, Thailand (REC.64-370-14-1). All patients voluntarily provided informed consent before participating in the study. The study was conducted under the ethical guidelines of the 1975 Declaration of Helsinki.

### Study population

We included all adult patients (age of at least 18 years old) with GI problems who visited the gastroenterology and hepatology outpatient clinic, and our endoscopic center between August and September 2021 (during the wave of delta variant of COVID-19 pandemic in Thailand) who voluntarily filled out the ADG questionnaire. Patients who had concurrent respiratory tract symptoms on the day of hospital visit and patients who reported to have other herbals or dietary supplements rather than ADG were excluded. In patients who had more than one visit during the study period, only the data from the first visit were included.

### Patient recruitment

All patients who visited the GI clinic, and the endoscopic center during the study period were invited to join the study. The ADG questionnaire (detailed as shown below) was distributed to all those who were invited along with the informed consent. The patients were allowed to take their time to decide whether they wanted to participate in the study. Those who voluntarily participated in the study signed the informed consent and filled the ADG questionnaire by themselves and handed it back to the nurses before entering the doctor’s room. Those who did not want to participate in the study brought the blank document back to the nursing staff, visited the doctor, and received their treatment as usual.

### Data collection

The questionnaire about ADG use is a single page questionnaire inquires whether the patient had consumed any herbal or dietary supplements aiming for health benefit within the past 30 days, and if so, what was that herbal or dietary supplement(s). In patients who reported having ADG within the past month, whether the product had been approved by Thai Food and Drug Administration (FDA), and how they consumed it (number of capsules or tablets per day and the duration of consumption) were inquired (the questionnaire in the original language is available in supplementary file 1). Other questions on the questionnaire included a history of self-SARS-CoV-2 infection, COVID-19 disease in family and close friends, how many shots of COVID-19 vaccination they had received at the time they filled out the questionnaire, their educational status, and monthly income.

Baseline demographic data of the patients i.e., age, sex, weight, height, underlying diseases, their GI conditions, and laboratory data including blood urea nitrogen (BUN), serum creatinine (Cr), and LFTs data regarding AST, ALT, alkaline phosphatase (ALP) at that visit and the prior visit (if available) were obtained from the hospital information system (HIS).

### Determine the change in liver enzymes

In order to evaluate the change in LFTs in patients who had ADG consumption compared with who did not, we only obtained the laboratory data of the prior visit if it fulfilled the following criteria: the timing of the LFTs was at least 28 days but no longer than 1.5 years prior to the current visit.

And for the analysis regarding changes in liver enzymes, the patients with known cause(s) of abnormal LFTs at the current or prior visit (according to their primary doctor’s note in medical records) e.g., hepatocellular carcinoma progression, hepatobiliary or pancreatic tumor, flare of underlying viral hepatitis B or C, or active autoimmune liver diseases were excluded.

The change in AST and ALT were reported as a delta change in the same person (the level at the current visit minus level at the prior visit), and as a pattern of change: increase, stable, and decrease, respectively. Using the reference of upper limit of normal of ALT at 25 U/L and 35 U/L in women and men, respectively^20^, we used the margin of 10% change from normal value (± 3 U/L from the prior visit) to be considered as a variation of ALT over time and categorized into the pattern ‘stable’. And those with delta ALT > 3 U/L and < 3 U/L were categorized into ‘increased’ and ‘decreased’ ALT groups, respectively.

### Statistical analysis

#### Sample size calculation

The sample size calculation was based on the sample size for a cohort study with binary outcome^21,22^. As there was no previous study regarding ALT change in patients who had ADG and control, we hypothesized that 30% of the patients who took ADG would have an increased ALT compared with 14% in patients who did not, and the proportion of patients who had ADG would be around 20% of study participants. A total of at least 390 patients (65 in the  ADG group and 325 in the control group) would be sufficient to demonstrate the result with a significance level of 0.05 and yield an 80% power.

#### Data analysis

All statistical analyses were performed using R program version 4.1.0 (Vienna, Austria). Descriptive statistics were used for baseline demographic data. Quantitative measurements were shown as mean ± SD or median with interquartile range (IQR) according to the distribution of observed values. The changes in laboratory data between the current visit and the prior visit within the same patients were analyzed using Wilcoxon signed-rank test. To compare between group of patients with and without ADG consumption, Chi-square test for categorical variables and Wilcoxon rank-sum test or t-test for continuous variables were used for the analyses. The univariable and multivariable logistic regression analyses were also carried out to identify independent factors associated with an increased in ALT level. A p-value of < 0.05 was considered statistically significant.

## Results

### Baseline characteristics

During the study period, 909 ADG questionnaires were filled, 23 were excluded as the questionnaires were filled by the same patients as they had frequent follow-up visits. Another 76 patients who reported to have herbal medications or dietary supplements other than ADG were also excluded. A total of 810 patients were included in this study. Of those, 168 patients (20.7%) had taken ADG within the past month. The baseline demographics, clinical characteristics, underlying co-morbidities as well as GI conditions in patients with and without ADG consumption (ADG group VS control group) were comparable, except for a higher proportion of male sex and a lower proportion of pre-existing liver cirrhosis were found in the ADG group than in the control group as shown in Table 1. When compared to the control group, patients in the ADG group had a significantly greater prevalence of having a history of SARS-CoV-2 infection either themselves (11% vs 6%) or in their close contacts (5.4% vs 1.9%).

**Table 1:** Baseline clinical characteristics between control group and ADG group in all 810 patients.

Variables	No ADG (n = 642)	ADG (n = 168)	P value
Age: median (IQR), years	59 (49.2,68)	56.5 (48.2,65.8)	0.138
Sex: male, N (%)	321 (50.1)	99 (59.3)	0.034
BMI: median (IQR), kg/m^2^	24.1 (21.7,26.8)	24.1 (21.8,27.4)	0.824
**Underlying comorbidities: N (%)**			
DM	136 (44.7)	26 (41.3)	0.614
HTN	171 (56.2)	36 (57.1)	0.897
CAD/CVD, on ASA	58 (19.1)	14 (22.2)	0.567
**GI/liver conditions: N (%)**			
Dyspepsia/GERD/IBS/constipation	107 (17.6)	38 (23.6)	0.083
Hepatitis	350 (57.6)	96 (59.6)	0.637
Cirrhosis	193 (31.7)	29 (18)	< 0.001
HCC	72 (11.8)	13 (8.1)	0.175
**Cause of liver diseases it they had hepatitis, cirrhosis, or HCC: N (%)**			
HBV	183 (45.9)	55 (54.5)	0.123
HCV	48 (12)	7 (6.9))	0.143
NAFLD	151 (37.8)	44 (43.6)	0.292
Alcohol	36 (9)	10 (9.9)	0.785
**COVID-19 related history: N (%)**			
Self-reported infected with COVID-19	38 (6)	18 (11)	0.026
Hx of COVID-19 in family or close friends	12 (1.9)	9 (5.4)	0.024
History of COVID-19 vaccination (**≥ **1 shot)	496 (77.6)	131 (78)	0.922
**Other demographic data: N (%)**			
Education level			0.722
- Elementary school	165 (26.8)	39 (24.8)	
- High school	149 (24.2)	36 (22.9)	
- College/Bachelor's degree	235 (38.1)	60 (38.2)	
- Master’s degree or Ph.D	67 (10.9)	22 (14)	
Income per month, median (IQR) Thai Baht	20,000 (10,000, 35,000)	24,500 (10,000, 40,000)	0.383

ADG, andrographolide; ASA, aspirin; BMI, body mass index; CAD/CVD, coronary artery disease/cardiovascular disease; COVID-19, Coronavirus disease 2019; DM, diabetes mellitus; GERD, gastroesophageal reflux disease; GI, gastrointestinal; IBS, irritable bowel syndrome; HBV, hepatitis B virus; HCC, hepatocellular carcinoma; HCV, hepatitis C virus; HTN, hypertension; IQR, interquartile range; N, number of patients; NAFLD, non-alcoholic fatty liver disease; Ph.D., Doctor of Philosophy.

Among patients who took ADG, 115 out of 168 (68.5%) of them reported to have Thai FDA-approved ADG products, while 31.5% reported that they take either non-Thai FDA-approved or did not know the Thai FDA approval status of the ADG products they had taken.

### Change in ALT in patients with and without ADG consumption

Of all 810 patients, 514 (63.5%) had available ALT data at two timepoints (current and prior visits) to compare. Twenty-nine patients who had definite causes of altered LFTs noted in medical records (e.g., hepatobiliary or pancreatic tumor, or flare of their viral/autoimmune hepatitis) were further excluded. Finally, a total of 485 patients were included in the LFT analysis, and the median duration of time difference in two LFTs was 160 (IQR: 84–192) days. There were 110 and 375 patients in the ADG and the control groups, respectively.

The median BUN, Cr, AST, ALT, ALP values among patients with and without ADG consumption at both time points, as well as the change (**Δ**) of those laboratory values in both groups of patients are shown in Table 2. In patients with available LFTs data to compare, around one-third of them had NAFLD at baseline, these proportions were similar in both groups.

**Table 2:** Comparison between control group VS ADG group in patients with available laboratory data (N = 486).

Variables	No ADG (n = 375)	ADG (n = 110)	P value
Presence of NAFLD at baseline: N (%)	125 (33.3)	35 (31.8)	0.766
Weight change: median (IQR), kg	0 (− 1,1)	0.4 (− 1,1.3)	0.100
BMI change: median (IQR), kg/m^2^	0 (− 0.4,0.4)	0.2 (− 0.4,0.5)	0.086
**At the current visit**			
BUN, median (IQR) mg/dL	12.4 (9.8,15.6)	11.9 (10,14)	0.290
Creatinine, median (IQR) mg/dL	0.9 (0.7,1.1)	0.9 (0.7,1)	0.255
AST, median (IQR) U/L	29 (23,39)	28 (23,37)	0.383
ALT, median (IQR) U/L	27 (19,39)	27.5 (19,39.8)	0.782
ALP, median (IQR) U/L	82 (67,107)	73 (60,92)	0.005
**At the prior visit**			
BUN, median (IQR) mg/dL	12.6 (10.2,16.3)	12.1 (10.1,14.8)	0.322
Creatinine, median (IQR) mg/dL	0.8 (0.7,1)	0.9 (0.7,1)	0.308
AST, median (IQR) U/L	28 (22,37)	26 (21,38)	0.136
ALT, median (IQR) U/L	27 (19,39)	23 (18.2,34.5)	0.360
ALP, median (IQR) U/L	81.5 (65,102)	68 (58.2,89.5)	0.001
**Change (Δ) in the same patient (level at current–prior visit)**			
Creatinine, median (IQR) mg/dL	0 (0,0.1)	0 (0,0)	0.350
AST, median (IQR) U/L	0 (− 5,5)	2 (− 3,8)	0.028
ALT, median (IQR) U/L	0 (− 5,5)	2 (− 3,8)	0.029
ALP, median (IQR) U/L	1 (− 5,8)	1 (− 5,6.5)	0.812
**ALT change group**			0.049
- Increase ALT level	121 (32.3)	49 (44.5)	
- Stable ALT level	146 (38.9)	38 (34.5)	
- Decrease ALT level	108 (28.8)	23 (20.9)	
ALT increase > 3 U/L, yes, N (%)	121 (32.2)	49 (44.5)	0.018

ADG, andrographolide; ALP, Alkaline phosphatase; ALT, Alanine aminotransferase; AST, Aspartate aminotransferase; BMI, body mass index; BUN, blood urea nitrogen; IQR, interquartile range; N, number of patients; NAFLD, non-alcoholic fatty liver disease.

There were significantly differences in the changes in AST and ALT between the two groups, patients in the ADG group had a median ALT change of + 2 (IQR: − 3,8) vs 0 (IQR: − 5,5) in the control group (p = 0.029). The same trend was also observed on the AST level (Table 2). And when categorized into the pattern of ALT change, a higher proportion of patients (44.5%) with ADG consumption experienced an ALT elevation > 3 U/L (increased ALT group) compared with 32.3% in patients with no ADG consumption (p = 0.018). Weight and body mass index (BMI) changes appeared to be numerically higher, although not statistically significant, in patients with ADG consumption as well.

We were also interested in the relationship between the dosage and duration of ADG consumption and ALT change; however, ADG products in Thailand were available in a wide range of andrographolide dosages per product unit and forms (e.g., capsule, tablet, powder, or even fresh leaves). As a result, it was not possible to convert the patient-reported comprehensive ADG intake data into a standardized unit or to determine if the dosage and duration of ADG consumption were related to changes in ALT.

**Figure 1:**
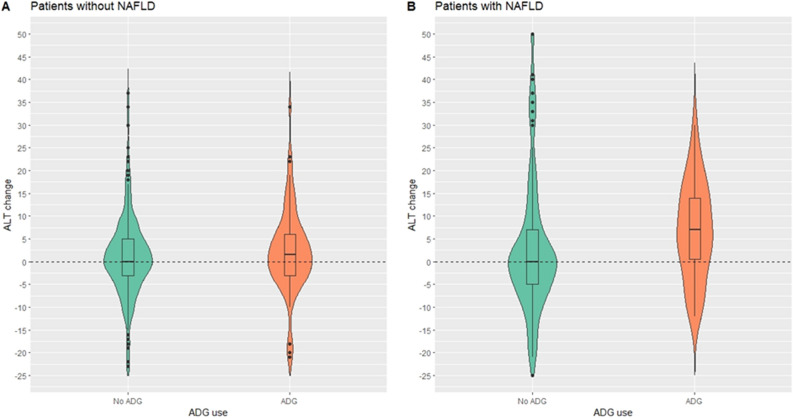
Violin plot of alanine aminotransferase (ALT) changes between *Andrographis paniculata* (ADG) VS control group stratified by non-alcoholic fatty liver disease (NAFLD) status. (**A**) Patients without NAFLD, and (**B**) Patients with NAFLD.

In both groups of patients, we looked at the impact of baseline NAFLD status on ALT elevation. According to the initial NAFLD status of the patients, Fig. 1 showed the difference in ALT levels between those who consumed ADG and those who did not. In patients without NAFLD at baseline (Fig. 1A), the ΔALT was similar in the ADG (median 0, IQR: − 4,5 U/L) and control groups (median + 1, IQR: − 3,6 U/L, p = 0.276). The ΔALT did, however, seem to be greater in patients who took ADG (median + 5, IQR: − 4,14 U/L) than in those who did not (median 0, IQR: − 7,7 U/L, p = 0.057) in those who had NAFLD at baseline (Fig. 1B).

### Factors associated with having an increased ALT

As mentioned earlier, a higher percentage of patients in the ADG group had elevated ALT levels (ALT > 3 U/L), but the ADG group also showed a greater increase in BMI than the control group. A multivariable logistic regression analysis was then used to determine whether ADG consumption was independently associated with increased ALT. When adjusted for age, sex, and the interaction between NAFLD status and BMI change, ADG was still independently associated with the development of ALT increased > 3 U/L with an adjusted odds ratio (OR) of 1.62 (95%CI 1.02–2.57, p = 0.042). While having NAFLD or having an increased BMI alone was not associated with an increased ALT, the interaction between having NAFLD and BMI was, with patients with NAFLD who had an increased BMI being more likely to have an increased ALT with an adjusted OR of 2.37 (95%CI 1.01–5.54, p = 0.046). The crude OR from univariable logistic regression analyses and the adjusted OR from a multivariable analysis are shown in Table 3. We also looked into whether having a history of COVID-19 was linked to a higher ALT because those who took ADG had higher rates of SARS-CoV-2 infection themselves. However, a univariable logistic regression found no evidence of a significant association between a history of COVID-19 infection and an elevated ALT (crude OR 1.55, 95%CI 0.78–3.07, p = 0.217).

**Table 3:** Logistic regression analyses of  predicting factors associated with an increased ALT (ΔALT > 3 U/L).

Factors	Univariable analysis Crude OR (95%CI)	Multivariable Adjusted OR (95%CI)	P value
Age, every 1-year increase	0.98 (0.96,0.99)	0.98 (0.96,0.99)	0.003
Sex, female vs male	0.74 (0.51,1.1)	0.77 (0.52,1.15)	0.198
ADG use, yes vs no	1.83 (1.17,2.85)	1.62 (1.02,2.57)	0.042
Presence of NAFLD, yes vs no	1.22 (0.81,1.82)	0.84 (0.48,1.46)	0.528
BMI increased, yes vs no	1.33 (0.9,1.96)	0.9 (0.55,1.48)	0.678
Presence of NAFLD and BMI increased, yes vs no	–	2.37 (1.01,5.54)	0.046

ADG, andrographolide; ALT, Alanine aminotransferase; BMI, body mass index; NAFLD, non-alcoholic fatty liver disease; OR, odd ratio.

## Discussion

In this study, we found that 20.7% of Thai patients had recently taken ADG during the COVID-19 pandemic aiming for their health benefit, but almost 90% of those who took ADG were not indicated as they did not have COVID-19. In the patients with available ALT data, we found that the patients in the ADG group had a significant increase in ALT compared to the patients in the control group (median + 2 (IQR: − 3,8) vs 0 (IQR: − 5,5), p = 0.029, respectively). And when using the threshold of ALT increased more than 10% of ULN (ALT change > 3 U/L) for the definition of ALT elevation, ADG use and patients with NAFLD who gained weight were independently associated with having ALT elevation with an adjusted OR of 1.62 (95%CI 1.02–2.57, p = 0.042) and 2.37 (95%CI 1.01–5.54, p = 0.046), respectively.

Under the current circumstance of COVID-19 pandemic which affects people globally and disrupts the public health systems in many countries, there are shortages in both healthcare facilities and modern antiviral medications, hence, an attention towards herbal medicine is increasing. The prevalence of herbal medicine use during COVID-19 situation varied across the different areas of the world, for examples, 25.8% from study in India^23^, 42% in Nepal^24^, and 57.6% in Bangladesh^25^. A public survey in Hong Kong showed 19.3% prevalence of Chinese herbal medicine used^26^. While a cross-sectional survey in Peru found a high prevalence of medicinal plants use up to 80.2% for preventing respiratory symptoms, and 71.0% for the treatment of respiratory symptoms during the COVID-19 pandemic^27^. Our study found that during the pandemic in Thailand, the prevalence of ADG usage in patients with GI disorders was 20.7%, which was comparable to the rate shown in Hong Kong but appeared to be lower than the studies conducted in the other countries previously mentioned. One possible explanation for this finding is that the patients in our study came from a gastroenterology and hepatology clinic, and more than half of them had chronic hepatitis or cirrhosis. Doctors will usually advise those patients to avoid taking any additional medications/ herbal products/ dietary supplements on a regular basis in order to reduce the risk of further hepatic derangement. We hypothesized that the finding of significantly lower proportion of cirrhotic patients seen in the ADG group compared with the control group could be justified by the same rationale. In addition, our findings of a higher proportion of having history of SARS-CoV-2 infection either themselves or in their family members and close friends in the ADG group than in control group were consistent with that observed in a study from Hong Kong^26^.

Being famously known as “King of bitters” which has many aforementioned properties for a long time, the effects of ADG on liver enzyme activity had not been systematically analyzed. There are reports advocated the hepatoprotective effect of ADG from the proposed mechanisms including against carbon tetrachloride (CCL_4_) and Tert-Butyl hydroperoxide (t-BHP), cytochrome P450 inducers effect, modulatory effect on Glutathione (GSH), anti-inflammatory activity or role in apoptosis pathway, etc.^4,5,28^ A recent in vitro study for anti-SARS-CoV-2 activity of ADG also affirmed that ADG has no cytotoxicity to six major human organs cell lines including hepatocytes^7^. And an in vivo study also demonstrates no significant changes in liver biochemistry of the mice after 21 days of andrographolide administration compared to controls^29^. However, when it comes to the ADG usage in humans in the real-world setting, the contradict results should not be negligible. In 2000, a phase I trial exploring the role of ADG in patients with human immunodeficiency virus (HIV) demonstrated that ADG usage led to significant rising of ALT levels with the mean rise of 109.3% at the 3rd week after initiation, and could return to baseline within 3 weeks after cessation^16^. Additionally, a recent multicenter randomized controlled trial in China including 130 patients also showed that 10.8% of patients in ADG group had increased ALT levels, and 13.8% had hyperbilirubinemia, compared to 10.8% and 7.7% in the control group, respectively^10^.

In the present study, we found that almost 90% of patients who took ADG had no history of SARS-CoV-2 infection, suggesting that there was some public misunderstanding about the use of ADG to prevent COVID-19, despite the fact that doing so was not advised. And those with ADG had significantly higher AST and ALT levels than those without ADG; a median ALT change of + 2 (IQR: − 3,8) in patients with ADG vs 0 (IQR: − 5,5) in patients without ADG (p = 0.029), and a median AST change of + 2 (IQR: − 3,8) in patients with ADG vs 0 (IQR: − 5,5) in patients without ADG (p = 0.028).

Despite the fact that the degree of ALT change was subtle (the median change was + 2 U/L), and there were no cases of severe hepatitis leading to hospitalization or mortality in our study, 44.5% of patients who took ADG had ALT elevation greater than 3 U/L, significantly higher than in patients without ADG (32.2%, p = 0.018). This may result in unnecessary investigations to determine the cause of abnormal ALT in those patients, as well as an increase in the cost of monitoring LFTs. Especially since the majority of patients took ADG to prevent SARS-CoV-2 infection, which ADG has no effect on, the misperception and misuse of ADG may add DILI burden to the already overburdened healthcare facilities.

Herbal medicine is increasingly being implicated as a cause of DILI. A recent systematic review and meta-analysis found that herbal and dietary supplements were the second most common agent classes causing DILI, with a prevalence of 25.3%, very close to the top rank of anti-tuberculosis agents (26.6%) in Eastern countries^30^. It is commonly believed among consumers that herbal medicines are made from natural products, and are therefore safer than conventional medications^31^. However, some herbal ingredients can exhibit hepatotoxicity themselves e.g., *Tinospora crispa*, and some of the herbal products may contain contaminants such as heavy metals during manufacturing processes and can lead to DILI^32^.

The mechanism of ADG use and ALT elevation is yet to discovered. In the present study, andrographolide can be in the form of compound, extract, or even traditional preparations. Formulations other than the pure compound itself will have different andrographolide and phytoconstituent content. The complex and rich nature of phytoconstituents, which varies with preparation methods/formulation in herbal medicine might lead to different pharmacological effects including toxicity. Moreover, the dosage, duration of consumption, or preparation forms of ADG might be associated with ALT change in ADG users. Due to a variety of amount, durations, and preparations of ADG use in our study, and there is no standardized protocol to calculate the andrographolide amount from a wide range of different ADG products in Thailand, a subsequent analysis regarding this issue cannot be made. Further studies are needed for a better clarification of the association between ADG and ALT change.

Additionally, as shown in Fig. 1, an increase in ALT in ADG users was more noticeable in patients with NAFLD than in patients without NAFLD, supporting previous research that NAFLD patients may be at risk of developing DILI. Nonetheless, we also concerned that ALT elevation in our patients could be from various conditions, therefore, we had excluded patients with known cause of ALT elevation such as tumor progression or flare of viral or autoimmune hepatitis, and patients reported to have other herbal or dietary supplements rather than ADG prior to the analyses of ALT change. In addition, during the COVID-19 pandemic, weight gain may be encountered in many patients^33^, and it might link to fatty liver and ALT elevation in a sequela. Taking this issue into consideration, we performed a multivariable logistic regression analysis of factors associated with the development of an increased in ALT (> 3 U/L), after an adjustment with age, sex, baseline NAFLD status, and whether the patients had an increased BMI, ADG use was still independently associated with ALT elevation with an adjusted OR of 1.62 (95%CI 1.02–2.57, p = 0.042).

Interestingly, neither having NAFLD status nor an increase in BMI alone was associated with an ALT elevation, but only patients with NAFLD who had BMI increased were independently associated with an increased risk of having ALT elevation for an adjusted OR of 2.37 (95%CI 1.01–5.54, p = 0.046).

The present study, to the best of our knowledge, included the largest number of patients comparing changes in ALT level between ADG and non-ADG users to date. The results of our study highlighted the association between the ADG usage and ALT elevation, an also demonstrated the significance of baseline NAFLD and weight gain in ALT elevation in this setting.

We acknowledged some limitations of our study, the causality of ADG causing DILI cannot be firmly concluded as the design of the study is rather cross-sectional and retrospective, and as mentioned earlier, there were different preparations, duration, and dosing of ADG used in our study making it impossible to identify whether Andrographolide compound itself or some ingredients from the different preparations of ADG that caused ALT elevation. And we did not record all medications the patients had taken regularly, as there might be some herb-drug interaction, or the use of other drugs may potentiate liver injury, however, we had excluded the effects of other herbal/ dietary supplements on the results of this study as 76 patients who reported to have herbal medications or dietary supplements other than ADG were not included in the LFTs analysis. Lastly, the interval between the first and second LFTs test among ADG and non-ADG users were varied and follow up LFTs after stopped ADG in patients who had ALT elevation were not performed, as well as rechallenge.

In summary, we found that at least one-fifth of the Thai patients taking ADG during the COVID-19 pandemic, in which most of them were misused. Patients with ADG consumption experienced more ALT elevation than in those without ADG, and patients with NAFLD who had gained weight were independently associated with an increased ALT > 3 U/L compared to baseline. The potential risk of ADG consumption on liver function should be further assessed in future studies with adequate documentation of detailed quality-related information on ADG preparations, to better establish a causal-relationship as well as the need for public precaution.

## Supplementary Information

Supplementary Information.

## Data Availability

The datasets generated and/or analyzed during the current study are not publicly available due to personal health information privacy policy but deidentification data are available from the corresponding author on reasonable request.

## References

[1] Ağagündüz D, Çelik M, ÇıtarDazıroğlu M, Capasso R (2021). Emergent drug and nutrition interactions in COVID-19: A comprehensive narrative review. Nutrients.

[2] Tallei TE, Niode N, Idroes R, Zidan B, Mitra S (2021). A comprehensive review of the potential use of green tea polyphenols in the management of COVID-19. Evid.-Based Complement. Altern. Med..

[3] Dai Y, Chen SR, Chai L, Zhao J, Wang Y, Wang Y (2019). Overview of pharmacological activities of *Andrographis paniculata* and its major compound andrographolide. Crit. Rev. Food Sci. Nutr..

[4] Chao WW, Lin BF (2012). Hepatoprotective diterpenoids isolated from *Andrographis paniculata*. Chin. Med..

[5] Chua LS (2014). Review on liver inflammation and antiinflammatory activity of *Andrographis paniculata* for hepatoprotection. Phytother. Res..

[6] Kumar RA, Sridevi K, Kumar NV, Nanduri S, Rajagopal S (2004). Anticancer and immunostimulatory compounds from *Andrographis paniculata*. J. Ethnopharmacol..

[7] Sa-Ngiamsuntorn K, Suksatu A, Pewkliang Y (2021). Anti-SARS-CoV-2 activity of andrographis paniculata extract and its major component andrographolide in human lung epithelial cells and cytotoxicity evaluation in major organ cell representatives. J. Nat. Prod..

[8] Enmozhi SK, Raja K, Sebastine I, Joseph J (2021). Andrographolide as a potential inhibitor of SARS-CoV-2 main protease: An in silico approach. J. Biomol. Struct. Dyn..

[9] Shi TH, Huang YL, Chen CC (2020). Andrographolide and its fluorescent derivative inhibit the main proteases of 2019-nCoV and SARS-CoV through covalent linkage. Biochem. Biophys. Res. Commun..

[10] Zhang XY, Lv L, Zhou YL (2021). Efficacy and safety of Xiyanping injection in the treatment of COVID-19: A multicenter, prospective, open-label and randomized controlled trial. Phytother. Res..

[11] Ervina M, Fadhil Pratama MR, Poerwono H, Siswodihardjo S (2020). The coronavirus disease 2019 main protease inhibitor from *Andrographis paniculata* (Burm. f) Ness. J. Adv. Pharm. Technol. Res..

[12] Pokhagul P, Kulsomboon W (2019). Signal detection of blood glucose lowering drugs and herbal medicines in the Thai databases on adverse drug events (Thai Vigibase). Thai J. Pharm. Pract..

[13] Benjaponpithak A, Visithanon K, Sawaengtham T, Thaneerat T, Wanaratna K (2021). Short communication on use of andrographis herb (FA THALAI CHON) for the treatment of COVID-19 patients. J. Thai Tradit. Altern. Med..

[14] Suwankesawong W, Saokaew S, Permsuwan U, Chaiyakunapruk N (2014). Characterization of hypersensitivity reactions reported among Andrographis paniculata users in Thailand using Health Product Vigilance Center (HPVC) database. BMC Complement. Altern. Med..

[15] Wechwithan S, Suwankesawong W, Sornsrivichai V, McNeil EB, Jiraphongsa C, Chongsuvivatwong V (2014). Signal detection for Thai traditional medicine: Examination of national pharmacovigilance data using reporting odds ratio and reported population attributable risk. Regul. Toxicol. Pharmacol..

[16] Calabrese C, Berman SH, Babish JG (2000). A phase I trial of andrographolide in HIV positive patients and normal volunteers. Phytother. Res..

[17] Teschke R, Danan G (2017). Drug-induced liver injury: Is chronic liver disease a risk factor and a clinical issue?. Expert Opin. Drug Metab. Toxicol..

[18] Lammert C, Imler T, Teal E, Chalasani N (2019). Patients with chronic liver disease suggestive of nonalcoholic fatty liver disease may be at higher risk for drug-induced liver injury. Clin. Gastroenterol. Hepatol..

[19] European Association for the Study of the Liver (2019). Clinical Practice Guideline Panel: Chair: Panel members, EASL Governing Board representative: EASL Clinical Practice Guidelines: Drug-induced liver injury. J. Hepatol..

[20] Terrault NA, Lok ASF, McMahon BJ (2018). Update on prevention, diagnosis, and treatment of chronic hepatitis B: AASLD 2018 hepatitis B guidance. Hepatology.

[21] Statistical Methods for Rates and Proportions, 3rd ed | Wiley. Wiley.com. (Accessed 5 Feb 2022) https://www.wiley.com/en-in/Statistical+Methods+for+Rates+and+Proportions%2C+3rd+Edition-p-9780471526292.

[22] Ngamjarus C, Chongsuvivatwong V, McNeil E (2016). n4Studies: Sample size calculation for an epidemiological study on a smart device. Siriraj Med. J..

[23] Charan, J. *et al.* Use of complementary and alternative medicine (CAM) and home remedies by COVID-19 patients: A telephonic survey. *Indian J. Clin. Biochem*. **36**(1), 108–111 (2020). 10.1007/s12291-020-00931-4 PMC7602770 33162692

[24] Khadka D, Dhamala MK, Li F (2021). The use of medicinal plants to prevent COVID-19 in Nepal. J. Ethnobiol. Ethnomed..

[25] Ahmed I, Hasan M, Akter R (2020). Behavioral preventive measures and the use of medicines and herbal products among the public in response to Covid-19 in Bangladesh: A cross-sectional study. PLoS ONE.

[26] Lam CS, Koon HK, Chung VCH, Cheung YT (2021). A public survey of traditional, complementary and integrative medicine use during the COVID-19 outbreak in Hong Kong. PLoS ONE.

[27] Villena-Tejada M, Vera-Ferchau I, Cardona-Rivero A (2021). Use of medicinal plants for COVID-19 prevention and respiratory symptom treatment during the pandemic in Cusco, Peru: A cross-sectional survey. PLoS ONE.

[28] Jayakumar T, Hsieh CY, Lee JJ, Sheu JR (2013). Experimental and clinical pharmacology of *Andrographis paniculata* and its major bioactive phytoconstituent andrographolide. Evid. Based Complement. Altern. Med..

[29] Bothiraja C, Pawar A, Shende V, Joshi P (2012). Acute and subacute toxicity study of andrographolide bioactive in rodents: Evidence for the medicinal use as an alternative medicine. Comp. Clin. Pathol..

[30] Low EXS, Zheng Q, Chan E, Lim SG (2019). Drug induced liver injury: East versus West—A systematic review and meta-analysis. Clin. Mol. Hepatol..

[31] Medina-Caliz I, Garcia-Cortes M, Gonzalez-Jimenez A (2018). Herbal and dietary supplement-induced liver injuries in the Spanish DILI Registry. Clin. Gastroenterol. Hepatol..

[32] Bunchorntavakul C, Reddy KR (2013). Review article: Herbal and dietary supplement hepatotoxicity. Aliment. Pharmacol. Ther..

[33] Clemmensen C, Petersen MB, Sørensen TIA (2020). Will the COVID-19 pandemic worsen the obesity epidemic?. Nat. Rev. Endocrinol..

