# Supplementary material for: The use of Andrographis paniculata and its effects on liver biochemistry of patients with gastrointestinal problems in Thailand during the COVID-19 pandemic: a cross sectional study
Source: Sci Rep. 2022 Oct 29;12:18213. doi: 10.1038/s41598-022-23189-7 (PMC9617865; doi:10.1038/s41598-022-23189-7)
Supplement: Supplementary file 1 — Supplementary Information. [file 41598_2022_23189_MOESM1_ESM.pdf]

แบบสอบถามการรับประทานยาสมุนไพรไทย และอาหารเสริม

ในผู้ป่วยโรคระบบทางเดินอาหาร โรงพยาบาลสงขลานครินทร์ ในช่วงที่มีการระบาดของโรคโควิค-19

1. ในช่วง 1 เดือนที่ผ่านมา ท่านได้รับประทาน ยาสมุนไพร อาหารเสริม สารสกัดจากธรรมชาติ เพื่อหวังว่าจะช่วยรักษาโรค หรือป้องกันโรค หรือเพื่อบำรุงสุขภาพ (หมายถึง ไม่ได้รับประทานเพื่อเป็นอาหาร) บ้างหรือไม่
   - ☐ รับประทาน (ไปข้อ 2)
   - ☐ ไม่ได้รับประทาน (ไปข้อ 4)
2. หากรับประทาน ท่านรับประทานสารชนิดใดต่อไปนี้บ้าง (เลือกได้ทุกข้อที่ท่านรับประทาน)
   - ☐ ฟ้าทะลายโจร ☐ มะรุม
   - ☐ กระชาย ☐ บอระเพ็ด
   - ☐ ถั่งเช่า ☐ คอลลาเจน
   - ☐ เฮอร์บาลไลฟ์ ☐ นิวตริไลท์
   - ☐ อื่นๆ ระบุ____
3. หากท่านรับประทาน ฟ้าทะลายโจร

   3.1 ชนิดของยาฟ้าทะลายโจรที่ท่านรับประทาน ผ่านการรับรองจาก อ.ย. หรือไม่

   ☐ ใช่ ☐ ไม่ใช่ ☐ ไม่ทราบ

   3.2 ท่านรับประทานยาฟ้าทะลายโจรอย่างไร

   ครั้งละ _________ เม็ด/แคปซูล วันละ __________ ครั้ง

   3.3 ท่านรับประทานฟ้าทะลายโจรมาแล้วนานเท่าไร (กะประมาณเอาเท่าที่ท่านจำได้)

   _____ ปี _________ เดือน _______ วัน
4. ท่านเคยได้รับการวินิจฉัยว่าติดเชื้อโควิด-19 หรือไม่
   - ☐ เคย ☐ ไม่เคย
5. เท่าที่ท่านทราบ มีคนในครอบครัว หรือคนใกล้ชิดของท่าน ติดเชื้อโควิด-19 บ้างหรือไม่
   - ☐ มี ☐ ไม่มี
6. ท่านได้รับการฉีดวัคซีนป้องกันโรคโควิด-19 แล้วหรือไม่
   - ☐ ฉีดแล้ว ☐ 1 เข็ม ☐ 2 เข็ม ☐ 3 เข็ม
   - ☐ ยังไม่ได้ฉีด
7. ระดับการศึกษาสูงสุดของท่าน
   - ☐ ประถมศึกษา ☐ มัธยมศึกษาตอนต้น ☐ มัธยมศึกษาตอนปลาย
   - ☐ ปวช. ☐ ปวส. ☐ ปริญญาตรี
   - ☐ สูงกว่าปริญญาตรีขึ้นไป ☐ ไม่ประสงค์จะแจ้ง
8. รายได้ต่อเดือน โดยเฉลี่ย _____________ บาท / เดือน หรือ ☐ ไม่ประสงค์จะแจ้ง

แบบสอบถามนี้เป็นเพียงการสำรวจเท่านั้น

<u>ไม่ได้มีคำแนะนำ</u>ให้ใช้ฟ้าทะลายโจร อาหารเสริม หรือสมุนไพรอื่น ในผู้ที่ไม่ติดเชื้อวิด-19 แต่อย่างใด
